# Integrating quality improvement, evidence-based practice, and knowledge translation into a health Sciences masters’ programme: a mixed methods study

**DOI:** 10.1186/s12909-025-07838-9

**Published:** 2025-10-14

**Authors:** Per Koren Solvang, Jan Egil Nordvik, Anders Vege, Hilde Tinderholt Myrhaug

**Affiliations:** 1 Department of Rehabilitation Science and Health Technology, Oslo Metropolitan University, Oslo, Norway; 2 Oslo Municipality, Oslo, Norway; 3 The Norwegian Health Directorate, Oslo, Norway; 4 Department of Nursing and Health Promotion, Oslo Metropolitan University, Oslo, Norway

**Keywords:** Quality improvement, Evidence-based practice, Knowledge translation, Implementation science, Improvement science, Master’s level teaching, Health care students

## Abstract

**Background:**

In recent years, the emphasis on quality improvement (QI), evidence-based practice (EBP), and knowledge translation (KT) in the education of health professionals has increased. Efforts have been made to identify commonalities between quality improvement and implementation science. However, the development of curricula that encompass these approaches remains poorly understood. Consequently, this study examines the implementation of a novel teaching module in quality improvement and evidence-based practice, designed for health sciences master’s students. The objective was to explore how students apply a combination of QI, EBP, and KT principles to enhance health services through clinically relevant, project-based assessments.

**Methods:**

The study utilised a mixed-methods approach. A survey assessed students’ self-perceived skills in quality improvement and the implementation of evidence-based practice. Of the 311 students enrolled, 163 (52%) participated in the pre-evaluation and 123 (39%) participated in the post-evaluation. Four focus group interviews with 24 students explored reflections on their learning process and the knowledge application within their respective professional fields. Project plans from 14 exam groups explored reflections on implementation. The qualitative data was analysed according to the principles of descriptive analysis.

**Results:**

Survey data indicated statistically significant improvements in seven student-reported learning outcomes from pre- to post-evaluation, specifically addressing issues such as setting up a literature search strategy and identifying relevant barriers and facilitators in planning an improvement project (*p* < 0.001). Learning outcomes were assessed as either confident or not confident. Focus groups and textual analysis identified three themes: teaching modelled as project consultation, improving with evidence, and qualifying for implementation work. Combined survey and qualitative data indicated that a supervised project exam is recommended. Integrating QI, EBP, and KT is advisable, but clarification of their integration is needed. Additionally, planning an improvement project for implementation in an existing organization is recommended as a framework for a project exam.

**Conclusions:**

The study supports a module for teaching quality improvement and implementation science within an integrated course for master’s students. Additionally, the study demonstrated the usefulness of a supervised continuous group examination task. Teachers adopting this course design should offer students a clear, robust, and explicit model for improving health services.

**Supplementary Information:**

The online version contains supplementary material available at 10.1186/s12909-025-07838-9.

## Background

In recent years, quality improvement (QI), evidence-based practice (EBP) and knowledge translation (KT) have been on the rise in educational programmes for health professionals [1]. QI provides a framework for assessing needs for improvement, planning and executing an intervention in a local context, evaluating whether changes have led to improved service delivery and patient satisfaction [2]. EBP secures the integration of the best available research, clinical experience, and patients’ preferences in clinical decision-making [3]. Finally, KT enables bridging the gap between research findings and their practical application in healthcare settings, ensuring that the latest evidence-based practices are quickly and effectively integrated into patient care [4]. In sum, both improvement science (QI) and implementation science (EBP and KT) are accepted as necessary in improving healthcare quality and safety [2].

Several studies have addressed the teaching of both improvement science and implementation science. A Swedish study on a master’s degree programme in QI reports positive effects on health services at the micro, meso and macro levels [5]. An updated umbrella review provides sound evidence of the positive effects of teaching EBP [6]. A review study of clinical and educational physician training interventions for QI reported successful clinical improvements [7]. However, there are calls for improved clarity in the teaching of improvement and implementation science.

A study on stakeholder attitudes towards a German programme underlines the need to combine implementation science and quality improvement in qualifying health service practitioners [8]. Studies of QI teaching modules in bachelor’s programmes for healthcare professionals call for clearer identification of which quality improvement competencies should be prioritised for students [9] and the need for curricular development in implementation science has been well-documented [10]. Glasziou et al. [11] conducted an analysis of the potential for integrating the two traditions and concluded: “Finally, those teaching the next generation of clinicians should value both disciplines, which should be taught, integrated and modelled in clinical training” (p. i16). These authors emphasise that healthcare quality and patient safety could significantly benefit if approaches such as quality improvement (QI), evidence-based practice (EBP) and knowledge translation (KT) were to inform one another. In alignment with this objective, several studies have identified potential synergies between improvement science and implementation science [2, 12–14]. Despite identified potential, the integration of these synergies remains underdeveloped in the teaching of healthcare quality improvement.

Moreover, recent work on the development of EBP suggests that the implementation phase remains underdeveloped, thereby hindering progress towards achieving high-quality services and improving patient safety [15]. While research shows that EBP teaching increases EBP knowledge among students and clinicians, it does not necessarily lead to EBP-related behaviours [16, 17]. Healthcare professionals’ willingness to adopt EBP behaviours may be negatively affected if their intentions to change are not sufficiently concretised, if no specific goals for improving care are established, or if positive feedback and consequences are lacking to reinforce sustained EBP practice [18]. Consequently, the know-do gap is identified as a significant issue: we possess knowledge, but we fail to act upon it. By combining QI (setting local goals) and KT (investigating barriers and facilitators) within the implementation of EBP teaching, students may enhance both their understanding and their capacity to engage meaningfully in the application of EBP. In an expert opinion article, Lehane et al. [19] concluded that “effectively embedding EBP throughout curricula requires further development, with a ‘real-world’ pragmatic approach that engenders dialogue and engagement with all stakeholders required” (p. 103).

The observed lack of clarity in the knowledge base, along with the limited real-world implementation of EBP, as noted in previous studies can be attributed to insufficient reflection on the diverse interpretations of improvement and implementation work, as well as inadequate emphasis on clinical practice implementation. Scholars maintain that approaches to improving health practices derived from improvement and implementation science often exist independently within both clinical services and in educational programmes [2, 20]. Consequently, there is a need for further studies on development of educational interventions that systematically integrate improvement science (QI) and implementation science (EBP and KT) while placing greater emphasis on implementation within clinical practice.

### Aim

The primary research aim was to understand if master’s level students experience learning to improve health services with the help of a teaching module based on an integration of QI, EBP and KT and with a clear emphasis on the implementation in clinical practice. This study investigated the extent to which the course altered students’ self-perceived skills in quality improvement and implementation science, and how they reflected on course design, learning process, and the impact on their abilities to improve practice.

## Methods

### Design

The study employed a mixed methods design to investigate the research question. Initially, a web-based survey was developed to evaluate students’ perceptions of skill improvement pre- and post-course completion. Subsequently, focus-group interviews were conducted to gain a detailed understanding of the learning process. Finally, to incorporate students’ examination projects into the qualitative analysis, the research team reviewed a sample of 14 examination papers. The analysis of these papers aimed to deepen comprehension of students’ learning outcomes.

An important aspect of the study’s design was addressing levels 2–4 in the Kirkpatrick model [21] of training outcomes [22]. The study evaluated what new knowledge was gained, which skills improved (Level 2), to what extent do students change their behaviour in their present and future work organisations (Level 3), and how do the education and training enhance work practices in an organisation (Level 4). Important to note, the skills element of level 2, level 3, and level 4 are elucidated by data concerning students’ self-reported *beliefs* about the course’s consequences for their future work. The survey data and examination papers offer self-reported outcomes related to Level 2. The focus group interviews provide insights into students’ self-reflections on all three levels of the model (Levels 2–4). In addition, the examination papers indicate a perceived potential for enhancing organisational practices, corresponding to Level 4.

The study’s reporting adhered to guidelines from the Mixed Methods Appraisal Tool (MMAT), version 2018 [23]. An overview is provided in Appendix 7.

### Setting

This article presents findings from a study of the implementation of a master’s level teaching module in quality improvement and implementation science at Oslo Metropolitan University. The module forms a mandatory component of the master’s level programme in health sciences that enrols professionals such as nurses, physiotherapists, and occupational therapists. Annually, approximately 300 students enrol in the programme. The students participate in one of 13 specialisations, detailed in Appendix 1. Half of the students undertake the quality improvement module in their first semester. The remaining half, following a part-time schedule over six semesters, engage with the QI module in their third semester. The module carries 10 credits in the European Credit Transfer and Accumulation System (ECTS), equivalent to 5 credits in the system used by universities in the United States.

### The implemented teaching module

The teaching of the module comprised of two key structuring elements: first, a systemisation of the three traditions – EBP, QI and KT – as outlined in Table 1. The development of Table 1 is based on studies that try to link different models of improvement and implementation science [2, 14, 24]. Teaching began with one week of full-day classroom sessions introducing the three approaches and examining the strengths and characteristics represented by each, as indicated in Table 1. For example, organisational change is most effectively addressed through the quality improvement approach, whereas the application of research knowledge to practice benefits significantly from the knowledge translation and evidence-based practice approaches [2]. In addition, separate sessions addressed user involvement, interprofessional collaboration, and strategies for literature search.

**Table 1:** Model of the three approaches applied in the implemented teaching module

	Quality improvement (QI)	Evidence-based practice (EBP)	Knowledge translation (KT)
*Origin*	Production industries outside the health sector (and applied to health services)	Systematising and applying knowledge as evidence for practice	Wanting to improve the ability of EBP to influence practice by implementation processes
*Problem identification*	Insufficient practice	Practice insufficiently based on the best evidence	Evidence not being implemented
*Dedication to organisational change*	Direct focus	Indirect focus	Indirect focus
*End-user involvement*	Clearly recognised	High priority in model	Recognised
*Sensitivity to context*	Clearly recognised	Highlighted in some versions	Clearly recognised
*Applying research knowledge to practice*	Recognised, but not a strong focus	Middle to strong relevance	Strong relevance
*Often used dual categorisation*	Improvement science	Implementation science	

The second structural element of the course involved an examination task where students outlined a quality improvement project, as detailed in Table 2. Students were required to identify a problem and suggest improving the situation (QI). They were also tasked with conducting a PICO literature search and critically appraising the selected systematic review or guideline (EBP). Furthermore, addressing user involvement was a mandatory part of the task (EBP). Ultimately, students were directed to create an implementation plan for the improvement, wherein the guideline or review article would play a pivotal role in substantiating the chosen intervention (KT). To strengthen the focus on the practical implementation of evidence-based practice, students were encouraged to use a case from clinical practice that was familiar to at least one group member, and particular emphasis was placed on identifying facilitators and barriers to implementation in the clinical context.

**Table 2:** Key elements^a^ of the examination assignment

Group examination with 3–6 students in each group. Maximum length of the assignment is 3,500 words.	
1. PROBLEM (NEED FOR IMPROVEMENT)	
• Begin with an example from a group member’s workplace or a familiar case that one or more of you are familiar with that requires improvement.	
2. PREPARATION	
• Identify relevant information and create an overview of your quality improvement topic. Include information that describes the need for improvement.	
• Describe the patients/service users, leaders and employee’s perspective of the quality improvement need.	
• Describe how user participation will be integrated into your quality improvement project.	
3. PLAN	
• Choose one quality improvement intervention that might be relevant to use for reaching your goal/outcome.	
• Prepare a clinical question using PICO (Problem – Intervention – Comparison – Outcome) based on your P, I and O.	
• Search for summarized research or clinical practice guidelines that may address your clinical question.	
• Based on your literature search, identify one scientific article or guideline that seems relevant to your clinical question. Describe the aim and the result of your selected article (chapter in clinical decision tool, systematic review, or guideline).	
• Based on a critical appraisal, discuss how the article or guideline support the implementation or give a reason for adjusting the implementation of the chosen intervention.	
• Discuss which other information sources should be considered in the decision-making of the quality improvement intervention and its implementation.	
4. IMPLEMENTATION	
• Describe a plan for the implementation of the intervention and the adoption of the new practice.	
• Describe strategies to ensure that all stakeholders involved in the QI project adhere to the new practice.	
• Identify barriers and facilitators within the implementation process.	

^a^Elements in the full assignment such as details on possible information sources in the preparation phase, recommended lay out, and review of group members’ involvement are omitted for simplicity

The introductory week of full-time classroom teaching concluded with a help desk session, where examination groups could ask initial questions about the assignment. Subsequently, each group was offered a 15-minute supervision session as the next step in the process. As a final stage, a mandatory presentation seminar was held, during which groups received feedback from both faculty and fellow students. The assignment was structured as a continuous group examination with 3–6 students per group. The exam paper was assessed on a pass/fail basis, guided by a grading template that outlined 18 key components of the assignment. To achieve a passing grade, students were required to satisfactorily meet at least 12 of these criteria. An overview of the course teaching and supervision can be found in Appendix 2.

### Data collection

Data collection involved three methods:

1. A digital survey was administered to students on the course’s digital learning platform at the start and end of the course, using a web-based questionnaire platform available to Norwegian universities (nettskjema.no). The students were asked seven questions about their skills (reported in Table 3).
2. Students were e-mailed and invited to participate in a focus group interview. After two weeks, four digital focus groups, each with seven students were recruited and scheduled for 1.5-hour sessions held in the early evening, conducted 6 weeks after course completion. Three students did not attend the focus group sessions, and one student withdrew from the study after the interview. Of the total 311 students enrolled in the programme, 172 were enrolled in full-time specialisations. Among the 24 students who participated in the focus groups, 15 were from full-time specialisations. The groups were assembled by the first author, ensuring representation from both part-time and full-time students, as well as from different specialisations within the programme. The first author moderated the interviews following a guide (Appendix 4), with assistance from the last author. The interviews, lasting between 60 and 80 min, were sound recorded and transcribed verbatim.
3. All students were e-mailed to seek their consent to include their examination papers in the study. There were 73 examination groups in total. After one round of reminders, all students in 14 examination groups consented, of which 8 groups were from full-time specialisations. The examination papers were obtained from the university examination administration. Further details on the recruitment of the students for interviews and assignments to use the examination papers are displayed in Appendix 1.

### Analysis

The survey’s pre- and post-evaluation survey data were unpaired due to respondent anonymity. Variables were ordinal (e.g. very confident, confident, not confident and not at all confident). These data were categorised as ‘confident’ and ‘not confident’ and were used as the dependent variables in our analysis. To investigate changes from pre- to post-evaluation, we conducted Mann–Whitney U tests in SPSS nr 28 with seven independent variables. A p-value of ≤ 0.01 was considered statistically significant.

The focus group interview transcripts and the examination papers were read and analysed according to the process suggested by descriptive analysis [25]. Descriptive analysis aims to remain close to participants experiences and expressions, aligning with the study’s objectives. However, thoroughness in developing themes is required. First, all authors read and familiarised themselves with the transcribed focus group interviews and examination papers. Codes for transcript sections were suggested and discussed in an initial meeting. The first author finalized the coding. The coded material was discussed at a subsequent analysis meeting with all authors, where initial themes were generated. Next, the first author devised a final suggestion for themes. Finally, all authors participated in refining and naming the final themes. An overview of coding examples and theme development is provided in Appendix 5.

## Results

### Student-reported skills – quantitative results

Of the 311 students enrolled in the course, 163 participated in the pre-evaluation and 123 participated in the post-evaluation, facilitated by the online data collection system Nettskjema.no, resulting in response rates of 52% and 39% respectively. Among the responders, 49.6% attended in their first master’s term, 5.7% in their second, and 43.1% in their third during the post-evaluation.

The pre- and post-analysis indicate statistically significant changes in students’ self-perceived skills during the course (Table 3).

**Table 3:** Student reported changes in skills before and after attending the course

Key learning goals Improvement approach(es) indicated	Pre data		Post data		*p*-value (Mann-Whitney U)
	Confident (n, %)	Not confident (n, %)	Confident (n, %)	Not confident (n, %)	
*1. Plan*,* execute and evaluate a quality improvement project (QI*^*a*^*)*	37 (22.7)	126 (77.3)	102 (82.3)	22 (17.7)	< 0.001* (4087.000)
*2. Fill in a PICO form (EBP* ^*b*^ *)*	106 (65)	57 (35)	119 (96)	5 (4)	<. 001* (6979.500)
*3. Set up a literature search strategy (EBP*,* KT*^*c*^, *QI)*	63 (38.7)	100 (61.3)	100 (80.6)	24 (19.4)	<. 001* (5862.000)
*4. Perform a search for relevant research literature (EBP*,* KT)*	88 (54)	75 (46)	100 (80.6)	24 (19.4)	<. 001* (7412.000)
*5. Critically appraise research papers (EBP*,* KT)*	75 (46)	88 (54)	96 (77.4)	28 (22.6)	< 0.001* (6932.000)
*6. Plan*,* execute and evaluate an implementation project (KT)*	24 (14.7)	139 (85.3)	95 (76.6)	29 (23.4)	<. 001* (3851.500)
*7. Identify relevant barriers and facilitators in an implementation project (QI*,* KT)*	32 (19.6)	131 (80.4)	105 (84.7)	19 (15.3)	<. 001* (3532.500)

*statistically significant results

^a^Quality Improvement

^b^Evidence Based Practice

^c^Knowledge Translation

Approximately half of the students found themselves confident in the tasks addressed before the course. After the course, approximately 80% of students reported confidence in achieving the course’s goals, and nearly all expressed confidence in completing a PICO form.

Students reported a significant improvement at a wide range of skills relevant to organising QI work, although with important nuances. When they entered the course, the students found themselves most confident in performing a search for relevant literature and critically appraising a research paper. For other QI specific learning goals, initial confidence levels were lower, resulting in a steeper learning curve. For instance, only 22% of students felt confident in planning a quality improvement project, but this figure rose to 82% after the course. A similarly steep learning curve was observed in identifying barriers and facilitators in implementation projects. No notable differences were observed between students who took the course in their first semester and those who completed it in their second or third semester. Overall, the findings suggest that, based on students’ self-reported beliefs, the development of skills in planning and executing improvement projects is a key outcome of the teaching module.

### Students’ reflections on course design, learning process and impact on their abilities to improve practice – qualitative results

A total of 24 students participated in the focus group interviews (Appendix 1). The data from the focus group interview and 14 exam papers were analysed and three main themes were developed (Appendix 5).

#### Teaching modelled as project consultation

The analysis identified three sub-themes in the students’ discussions on teaching: course pedagogy, learning paths, and the integration of improvement models. Together, these sub-themes highlight how the teaching was experienced as a form of project consulting. Students received an integration of improvement models, expert advice on project development, and noted that their skill levels influenced the preferred advisory strategies during supervision.

Course pedagogics were addressed in all group discussions. Some discussions were categorised as internal to the course evaluation and omitted here (e.g., the pros and cons of digital teaching and the organisation of examination groups). As outlined above, the course was organised with one week of full-day classroom teaching, ending with a help desk where examination groups could ask initial questions about the assignment. Later, the examination groups were offered a 15-minute supervision session as the next step. As a final stage, a mandatory presentation seminar was organised in which the examination groups received advice from staff and fellow students. The students unanimously praised the teaching approach as exemplified by participants in Focus group (FG) 2.

*I got more out of working on the exam task in the group than teaching in plenary sessions*.

(Student (S) 4 (speakers’ id number in interview transcript))

“*Yes. In hindsight*,* I agree that working on the exam task was where I learned the most.” (S5)*.

The project-based organisation of the teaching was recognised as helpful for enhancing competence in improvement and implementation work. When evaluating input from teachers, the supervision pathway was praised.

*“I have to say that I think it was really good that you were able to have that kind of guidance along the way where we could have a conversation with the teacher because*,* like*,* are we really on the right track? And then we kept on working*,* and later we had a new guidance where we said something about how we had been doing this since the last time.” FG2*,* S1*.

“*I also think listening to the others’ guidance was good*,* and it was nice that they also listened to ours. It was instructive*,* and we got reassured that the challenges we had*,* they had the others as well*,* and then we were able to identify common issues that we got good answers to.” FG2*,* S4*.

Students found that both working on their own project plans and participating in a joint group of projects facilitated their learning effectively. Students noted that the type of guidance provided in this course was rare in other university courses.

The students also addressed the models of improvement applied in the course. From the student perspective, addressing the combination of models, the opinions were divergent. Some students wanted to relate to one clear-cut model.

*“I can say that when we found the website that the Norwegian Institute of Public Health has on that quality improvement model and read it*,* we were like*,* yes*,* that’s the whole task*,* and that point-by-point what we’re going to do. And then we talked about how frustrating it was in class that you were led through many different models. Why couldn’t you refer to this one? It would have become clearer in a way*,* we thought.” FG2*,* S7*.

In contrast, other students appreciated the introduction to multiple models.

*“I think it was also really good with quality improvement systematised. And that table systematising the models for improvement. You have heard about quality improvement*,* but here it was more structured. I thought that was nice and helpful.” FG3*,* S8*.

Students who favoured a systematic approach incorporating multiple models (as outlined in Table 1) were typically in their third semester (of six). The student who preferred a single -model approach was, along with her examination group, in her first semester. Hence, the level of experience may have influenced how the students related to merging versus simplicity in the approach chosen for the course. The placement of the course in the study plan was also directly addressed in two interviews. Students who took the course in their third semester expressed satisfaction with the placement.

*“We talked about it a little bit in my examination group because we’ve gone a year first with the philosophy of science and research methods*,* and that’s an advantage versus starting straight into this course in your first semester. You should know a little bit about confidence intervals and some p-values and stuff like that. I think it was helpful that we have the background I have then – since I’m now in my third semester.” FG4*,* S7*.

Students were clear about the value of the course’s project consultation format, which effectively channelled teaching resources into supervising examination project work. They also agreed that the ideal placement of the course, if it involved merging improvement models, was between the second semester and the start of work on the master’s thesis in the third or fourth semester. To gain a deeper understanding of the challenges brought up, a closer look at the exam assignment and the examination papers handed in will be given in the next section.

#### Improving with evidence

The analysis, as detailed in the Methods section and in Appendix 5, identified two sub-themes in evidence-based improvement. The first sub-theme identifies how exam group projects align with the components of the combined improvement model (Table 1). The second sub-theme identifies the connection between improvement needs and evidence.

In terms of problem identification, the examination papers primarily adhered to the QI tradition. Students selected cases based on relevant issues within their specialisations. Appendix 6 lists the projects included for analysis. They are thematically representative of the entire set of 68 projects. Most papers aimed to improve insufficient practices, with some exemptions, such as the paper on implementing the Olweus anti-bullying programme. This examination paper exemplifies the core logic from KT, where evidence exists but has not been implemented.

For organisational change, direct attention was required in the implementation section of the assignment. However, indirect attention to organisational change, a hallmark of EBP, characterised the examination papers. End-user involvement was commonly a weak part of the examination papers. Students struggled to involve clients at the meso level by including representatives in planning and execution. Some papers defined professionals as users, whiles others included knowledge from studies on patient experiences relevant to the improvement project.

Regarding sensitivity to context, this aspect was strong in the papers based on a case from the workplace of one of the group members. The trend in the exam papers was to clearly acknowledge context, consistent with the QI approach. Finally, the application of research knowledge to practice was explicitly required in the examination task, and all exam papers identified relevant evidence-bases for their projects. However, the papers differed widely in the degree to which they made a connection between the evidence and the intervention to be carried out. By this measure, the papers kept distance to the KT approach.

The relationship between interventions and evidence in quality improvement work is complex. This complexity is heightened when combing the three traditions, as outlined in Table 1. This challenge was highlighted in one focus group interview.

*“In terms of the assignment text*,* I thought it was good and educational*,* but it had maybe a bit of a weird structure. Moreover*,* when we received advice on the examination paper*,* we were told that we should have our quality improvement intervention ready before the PICO. And then you get down to the PICO*,* and you must come up with something. It felt like it was sort of in the wrong order.” FG2*,* S6*.

The challenge addressed by this student on behalf of her group can be interpreted as a result of the competing logics of QI, EBP, and KT. In QI, the evidence base is less prominent, with the main focus on identifying problems and proposing solutions based on local resources. In KT and EBP, the primary focus is on the clinical application of research findings, with these findings playing a central role. This interpretation indicates challenges in implementing a combination of the three traditions. Further support for this interpretation came from students, as cited in the previous section, who advocated for the exclusive application of the PDSA cycle, a model significant to QI. These challenges can be understood as depending on the relationships between problem, measure, and evidence. The identified need for improvement, suggestions for enhancing practice, and the evidence supporting improvement interventions are all critical elements in quality improvement work. However, the relationship between these elements is not entirely clear. The most challenging relationship is between designing an improvement and its evidence base. In some cases, the intervention is closely linked to evidence, such as anti-bullying programmes developed alongside clinical trials. In other cases, interventions are based on more generalised support from systematised research, as seen in the paper that uses checklists to improve adherence to clinical guidelines (Appendix 4).

#### Qualifying for implementation work

Two sub-themes emerged from the analysis of the students’ reflections on the meaning of the course for their position as employees in health services. The course’s significant emphasis on the implementation phase was the first issue. The second issue was that enhanced competence in quality improvement was crucial for regular participants in quality improvement projects. These issues together indicated that implementation competence as the core outcome of the course.

EBP and the related approaches of QI and KT were known to students. Most students had at least a few years of clinical practice, and many had been exposed to these improvement models from the start of their bachelor’s programme up to the beginning of their master’s programme. However, they found that the impact of these approaches on actual health practice was often unclear.

*“I think many have heard about and become tired of evidence-based practice because we’ve been getting it in with a teaspoon since day one*,* but this course is excellent because it focuses on the implementation. It dawns on us how to use evidence-based practice in clinical work. For many*,* it can be an empty term*,* so it was nice that you lifted the practice element. I think it was a wake-up call for some. Thinking*,* ‘Yes*,* that is how we can work with evidence-based practice.” FG3*,* S5*.

Students also highlighted that the course design, which included project planning as an examination task, effectively facilitated the transfer of learning into clinical practice.

*“I felt that the implementation was the most important thing. Being updated on research is emphasised in many contexts*,* but here*,* we had the opportunity to work with a process where the actual implementation is to be carried out. It was beneficial and a strength in the course.” FG1*,* S5*.

The examination task, which was central to the course, was perceived as meaningful by both students familiar with improvement work and those with limited experience in it. All students felt that they gained competence in improving practices within their current or future occupational roles and organisations.

The main aim of the course was to enable students to apply their knowledge and skills in managing and executing quality-related work. Students emphasised that their goal was not necessarily to lead improvement processes but to become highly skilled participants.

*“This course has a huge relevance. Even if you are not directly involved in quality-enhancing work in your role or do not see it your daily work*,* you are part of it as an employee. It’s about understanding the rationale behind it. A better understanding of why we work the way we do fosters improved collaboration in practice. When this logic is not attributed to management or individual people in a workplace*,* we can spread that expertise.” FG3*,* S6*.

The non-leading role in improvement work involves two elements. First, it is about being an active and conscious participant in a quality improvement project. Second, the course fosters a continual awareness that working with evidence-based methods is integral to clinical practice in health care.

## Discussion

To summarise the findings, results from the mixed methods approach are integrated in Table 4. First, it became evident that the project planning aspect of the teaching module led to a self-reported steep learning curve, where project consulting was the primary teaching approach. Secondly, while students were familiar with evidence finding, the qualitative analysis demonstrated that linking improvement needs and evidence was challenging. Finally, implementing an improvement idea within an existing organisation appeared to give students confidence in both initiating and taking part in improvement work.

**Table 4:** Linking the study results and providing conclusions

Improvement task	Learning curve based on survey data	Themes developed from qualitative data	Merged take home messages
*Planning*	Steep (Q1, Q6)^a^	Teaching modelled as project consultation	Supervised project exam recommended
*Finding evidence*	Sleek (Q2, Q3, Q4, Q5)	Improving with evidence	QI, EBP and KI trifecta recommended but clarified fusion needed
*Implementing*	Steep (Q1, Q6, Q7)	Qualifying for implementation work	Implementation in existing organisation recommended as exam task

^a^Question numbers in Table 3

The students reported significant improvements in crucial learning outcomes (Table 3), although several challenges were identified in the analysis of the qualitative material. The balancing between suggesting quality improvement interventions and finding scientific backing, on the one hand, and pointing out research-based knowledge in need of better implementation, on the other, was addressed. Applying research evidence is not strongly focused in the QI approach [2]. This lack of focus is a possible explanation for the identified weaknesses in applying scientific evidence when analysing the examination papers.

In the examination papers, students struggled with user involvement at the organisational meso level during project planning. User involvement in clinical encounters at the micro level is well-known to most health practitioners [20]. This type of user involvement was also reflected in the examination papers, even if not explicitly asked for. User involvement at the organisational meso level may be less familiar to health practitioners [26]. A possible explanation is that the goal nearest at hand for nurses and other healthcare professionals is to improve the output of their work. As a result, meso level patient involvement is often secondary. The study context may exacerbate this trend. In the university setting, students work among themselves, without direct patient involvement. The lack of end user involvement can also be seen as representing a weakness in the improvement models. Involvement of patients and next of kin is clearly addressed as part of the EBP approach, but the involvement of representatives on the organisational meso level is not well clarified in any of the models.

Previous studies have highlighted the need for better theoretical foundations in teaching improvement and the implementation of scientific evidence in clinical practice [9]. Scholars have pointed to the possible value of bridging the silos between the improvement and implementation sciences [2, 11]. In this study of a master’s level course, the differing approaches were clarified through the systematisation outlined in Table 1. Most of the students welcomed this systematisation. However, challenges arose when applying this systemisation to the actual design of improvement projects. This challenge suggests a possible weakness in clarifying the scholarly foundation for improvement. The students praised the systematisation because it clarified the relationship between approaches they had previously been introduced to. However, it is essential to question whether the clarification of the base for combining improvement approaches has gone far enough. Both the examination task and the name of the course highlighted quality improvement. Thus, EBP and KT tend to play a lesser role. An important goal for further development of combining strengths in related improvement approaches and curricular studies, is to reflect on the possibility of clearly formulating an approach that takes on board the best of the three approaches of improvement work and develops clearer guidelines that build on the identified strengths.

An important lesson from the current study was the positive evaluation of prioritising group work advising over traditional classroom teaching. This echoes the findings from a study of a comparable teaching module for medical students, in which proposing a QI intervention was an essential learning goal. This study highlighted the benefits of explicit instruction and mentorship [27].

In the framework of Kirkpatrick’s [21] four levels of training outcomes, the students self-responded to the knowledge they had gained after completing the course and the skills that had improved in the focus group interviews (level 2). Skills improvement was also addressed in the analysis of the examination papers. The emerging pattern indicated that students believed that they significantly improved their skills in planning improvement projects. The students also addressed the third level and fourth level: how they reported changed behaviour in the workplace (level 3) and how they envisaged changes in how the organisation would be affected (level 4). Upon completing the course, they regarded themselves as competent participants in improvement work. This experience also makes the process of subjectivation introduced by Biesta [28] relevant. The students self-reported a new sensibility for improvement work and anticipated contributing to a stronger focus on improvement in their clinical settings.

### Strengths and limitations

The survey response rate was 52 pre-course and declined to 39 post-course, and the two data sets are not linked. Another significant limitation is that much of the data comprises self-reported perceptions of teaching outcomes, which substantially limits the ability to demonstrate actual course impact. Additionally, the survey did not collect data on participants’ prior clinical experience, which may have influenced their learning outcomes. The textual analysis of examination papers provides independent data; however, it does not evaluate actual changes in workplace behaviour. Another limitation is that the interview moderators and authors of the paper are also the course instructors. This may introduce a bias towards highlighting positive experiences with the course. Yet, the authors have taken great care in producing and handling data to meticulously evaluate and improve the course.

However, the study has some important strengths. It examines a teaching intervention that combines the important elements of improvement science and implementation science. This makes the study a novel contribution to the literature on improving teaching to healthcare students. Secondly, the study employs a triangulation method, incorporating a survey, group interviews, and text analysis. While the findings are based on self-reported experiences within a learning context, the use of diverse data sources provides a broad and empirically grounded understanding of how students engaged with and experienced the innovative course format.

## Conclusion

The study suggests that incorporating a broad base of improvement and implementation science for teaching improvement is effective with health science master’s students. Teachers of master’s courses with a similar scope are advised to move beyond merely bridging approaches. A systematised and clearly defined approach will be most beneficial for students. Moreover, the results indicate that students found a continuous group examination task supervised, at the expense of auditorium teaching, to be useful.

Several studies suggest that combining the strengths of approaches such as QI, EBP, and KT is highly beneficial. Further research is needed to explore how such combinations can be implemented in educational and clinical contexts.

## Supplementary Information

Supplementary Material 1: Appendix 1. Overview of Specialisations and Recruitment to Interviews and Access to Examination Papers.

Supplementary Material 2: Appendix 2. Course Overview.

Supplementary Material 3: Appendix 3. Survey Questions

Supplementary Material 4: Appendix 4. Interview Guide for Focus Groups.

Supplementary Material 5: Appendix 5. Overview of the Qualitative Analysis Process.

Supplementary Material 6: Appendix 6. Aims in Student Projects Made Available for Analysis.

Supplementary Material 7: Part I. Mixed Methods Appraisal Tool (MMAT), version 2018.

## Data Availability

The survey data supporting the findings of this study are available from the corresponding author, upon reasonable request. The interview and textual data are not available, as participants did not give written consent for public sharing.
