# Supplementary material for: Integrating quality improvement, evidence-based practice, and knowledge translation into a health Sciences masters’ programme: a mixed methods study
Source: BMC Med Educ. 2025 Oct 14;25:1420. doi: 10.1186/s12909-025-07838-9 (PMC12522345; doi:10.1186/s12909-025-07838-9)
Supplement: Supplementary file 1 — Supplementary Material 1: Appendix 1. Overview of Specialisations and Recruitment to Interviews and Access to Examination Papers. [file 12909_2025_7838_MOESM1_ESM.docx]

**Appendix 1: Overview of Specialisations and Recruitment to Interviews and Access to Examination Papers**

| ***Specialisation*** | ***Number of enrolled students*** | ***Students in focus group interviews*** | ***Examination papers*** |
| --- | --- | --- | --- |
| *Empowerment and Health Promotion* | 26 | 3 | 2 |
| *Rehabilitation and Habilitation* | 19 | 3 | 1 |
| *Nutrition Competencies for Health Personnel* | 20 | 1 | 0 |
| *Occupational Therapy* | 7 | 0 | 0 |
| *Physiotherapy for Children and Adolescents* | 23 | 1 | 1 |
| *Physiotherapy for Older Adults* | 22 | 1 | 1 |
| *Physiotherapy for Musculoskeletal Health* | 29 | 1 | 2 |
| *Psychomotor Physiotherapy* | 23 | 1 | 1 |
| *Public Health Nursing* | 79 | 7 | 5 |
| *Cancer Nursing* | 16 | 4 | 1 |
| *Nursing – Clinical Research and Professional Development* | 16 | 2 | 0 |
| *Public Health Nutrition* | 31 | 0 | 0 |
| *Total number of students* | 311 | 24 | 14 |
