# Supplementary material for: Integrating quality improvement, evidence-based practice, and knowledge translation into a health Sciences masters’ programme: a mixed methods study
Source: BMC Med Educ. 2025 Oct 14;25:1420. doi: 10.1186/s12909-025-07838-9 (PMC12522345; doi:10.1186/s12909-025-07838-9)
Supplement: Supplementary file 2 — Supplementary Material 2: Appendix 2. Course Overview. [file 12909_2025_7838_MOESM2_ESM.docx]

**Appendix 2: Course Overview**

| *Weeks* | 1 | 2 | 3 | 4–5 | 6–7 | 8 |
| --- | --- | --- | --- | --- | --- | --- |
| *Plenary teaching and exam* | Classroom teaching |  |  |  |  | Exam paper deadline |
| *Supervision of exam groups* | Help desk for choosing a project |  | Supervision sessions with 15-minute slots Comments by   - Course team member - Specialisation representative - Research Librarian |  | Presentation seminars with 30-minute slots Comments by   - Course team member - Specialisation representative - Peer group |  |
