# Supplementary material for: Integrating quality improvement, evidence-based practice, and knowledge translation into a health Sciences masters’ programme: a mixed methods study
Source: BMC Med Educ. 2025 Oct 14;25:1420. doi: 10.1186/s12909-025-07838-9 (PMC12522345; doi:10.1186/s12909-025-07838-9)
Supplement: Supplementary file 3 — Supplementary Material 3: Appendix 3. Survey Questions [file 12909_2025_7838_MOESM3_ESM.pdf]

Appendix 3

# Questionnaire before taking MAVIT4100 Quality Improvement and Implementation of Evidence-Based Practice

Mandatory fields are marked with an asterisk *

What term are you in of your master's degree? *

- ○ 1
- ○ 2
- ○ 3
- ○ 4
- ○ Not doing a Master

How confident do you feel about performing the following activities on your own?

| | Very confident | Confident | Not confident | Not at all confident |
|---|---|---|---|---|
| Plan, execute and evaluate an quality improvement project * | ○ | ○ | ○ | ○ |
| Fill in a PICO-form * | ○ | ○ | ○ | ○ |
| Set up a literature search strategy * | ○ | ○ | ○ | ○ |
| Perform a search for relevant research literature * | ○ | ○ | ○ | ○ |
| Critically appraise research papers * | ○ | ○ | ○ | ○ |
| Plan, execute and evaluate an implementation project * | ○ | ○ | ○ | ○ |
| Identify relevant barriers and facilitators in an implementation project * | ○ | ○ | ○ | ○ |

Previous experience and knowledge

| | Yes | No | Not relevant/do not know |
|---|---|---|---|
| Do you have any prior experience in quality improvement projects from? | ○ | ○ | ○ |
| Do you think this course is relevant for you specialization? | ○ | ○ | ○ |
| Do you think this course will be relevant in your future career? | ○ | ○ | ○ |
| Do you think user involvment and updated evidence are critical in clinical practice? | ○ | ○ | ○ |

# Course Evaluation Form for MAVIT4100 Quality Improvement and Implementation of Evidence-Based Practice

Mandatory fields are marked with an asterisk *

What term are you in of your master's degree? *

- ○ 1
- ○ 2
- ○ 3
- ○ 4
- ○ Not doing a Master

## Quality of the learning resources

To which extent were the following activities helpful for your learning outcome?

| | Very helpful | Helpful | Not helpful | Not at all helpful | N/A - Did not participa-te/use |
|---|---|---|---|---|---|
| CANVAS - Text describing the course and the course modules * | ○ | ○ | ○ | ○ | ○ |
| CANVAS - Videos and pre-recorded lectures * | ○ | ○ | ○ | ○ | ○ |
| Lectures at Zoom/campus * | ○ | ○ | ○ | ○ | ○ |
| Help desk at Zoom * | ○ | ○ | ○ | ○ | ○ |
| Seminars at Zoom/campus * | ○ | ○ | ○ | ○ | ○ |
| The group-based exam * | ○ | ○ | ○ | ○ | ○ |
| Direct contact with course organizers (e.g. by e-mail og messages in CANVAS) * | ○ | ○ | ○ | ○ | ○ |

Overall, how satisfied are you with the teaching and the outline of the course? *

- ○ Very satisfied
- ○ Satisfied
- ○ Neither
- ○ Dissatisfied
- ○ Very dissatisfied

## What did you learn?

How confident do you feel about performing the following activities on your own?

| | Very confident | Confident | Not confident | Not at all confident |
|---|---|---|---|---|
| Plan, execute and evaluate an quality improvement project * | ○ | ○ | ○ | ○ |
| Fill in a PICO-form * | ○ | ○ | ○ | ○ |
| Set up a literature search strategy * | ○ | ○ | ○ | ○ |
| Perform a search for relevant research literature * | ○ | ○ | ○ | ○ |
| Critically appraise research papers * | ○ | ○ | ○ | ○ |
| Plan, execute and evaluate an implementation project * | ○ | ○ | ○ | ○ |
| Identify relevant barriers and facilitators in an implementation project * | ○ | ○ | ○ | ○ |

### Learning outcomes - Knowledge

To what extent do you think that the course has contributed to the attainment of the following knowledge:

| | A very large extent | A large extent | Quite large extent | Minor extent |
|---|---|---|---|---|
| Can describe the health and care services' management, organisation and framework conditions * | ○ | ○ | ○ | ○ |
| Can explain what characterises a high-quality service * | ○ | ○ | ○ | ○ |
| Can discuss prioritisation criteria and ethics in the health and care services * | ○ | ○ | ○ | ○ |
| Can explain management strategies in innovation and implementation of evidence-based practice * | ○ | ○ | ○ | ○ |
| Has insight into the UN Sustainable Development Goals, particularly Goal 3, Good Health and Well-being, and an understanding of the connection between health and sustainable social development * | ○ | ○ | ○ | ○ |

### Learning outcomes - Skills

To what extent do you think that the course has contributed to the attainment of the following skills:

| | A very large extent | A large extent | Quite large extent | Minor extent |
|---|---|---|---|---|
| Can apply models for quality improvement (preparation, planning, execution, evaluation and implementation) * | ○ | ○ | ○ | ○ |
| Can apply different quality indicators to analyse quality improvement projects * | ○ | ○ | ○ | ○ |
| Can contribute to quality improvement on the basis of relevant knowledge, research and user participation * | ○ | ○ | ○ | ○ |
| Can provide structured feedback and assessments to fellow students on quality improvement work in progress * | ○ | ○ | ○ | ○ |
| Can carry out systematic knowledge searches related to specific research question * | ○ | ○ | ○ | ○ |

### Learning outcomes - General competence

To what extent do you think that the course has contributed to the attainment of the following general competence:

| | A very large extent | A large extent | Quite large extent | Minor extent |
|---|---|---|---|---|
| Can apply knowledge and skills in managing and executing quality-related work, including implementing evidence-based practice * | ○ | ○ | ○ | ○ |
| Can contribute to interdisciplinary cooperation in the quality improvement of health and care services at the individual, service and societal level * | ○ | ○ | ○ | ○ |
| Can contribute to the phasing-out and implementation of methods and technology intended to improve the quality of services * | ○ | ○ | ○ | ○ |

How can user participation be promoted in quality improvement projects? Respond in English or Norwegian

Do you have any written feed-back to the course organizers? Respond in English or Norwegian
