# Supplementary material for: Integrating quality improvement, evidence-based practice, and knowledge translation into a health Sciences masters’ programme: a mixed methods study
Source: BMC Med Educ. 2025 Oct 14;25:1420. doi: 10.1186/s12909-025-07838-9 (PMC12522345; doi:10.1186/s12909-025-07838-9)
Supplement: Supplementary file 4 — Supplementary Material 4: Appendix 4. Interview Guide for Focus Groups. [file 12909_2025_7838_MOESM4_ESM.docx]

**Appendix 4: Interview Guide for Focus Groups**

How have you experienced working with the teaching module?

- The examination paper
- Working in groups
- The connection between different parts of the course
- The teaching
- The resources in the digital learning platform
- Reading list
- Other things

How has MAVIT4100 contributed to the specialist knowledge you have as professionals?

- How well informed were you about quality improvement work before starting the course?
  - - Quality improvement (the PDSA cycle and the model developed by the Norwegian Institute of Public Health)
    - Knowledge-based practice
    - Knowledge translation
- Have you gained new ideas on how you can contribute to the development of your own workplace?
- Will you continue to keep up-to-date about quality improvement work and implementation of knowledge-based practice?
- Do you have any ideas on how the course could improve in translating knowledge to practice?
- Other things

How could the course be better facilitated for developing master’s theses in quality improvement?

Do you have other input?
