# Supplementary material for: Integrating quality improvement, evidence-based practice, and knowledge translation into a health Sciences masters’ programme: a mixed methods study
Source: BMC Med Educ. 2025 Oct 14;25:1420. doi: 10.1186/s12909-025-07838-9 (PMC12522345; doi:10.1186/s12909-025-07838-9)
Supplement: Supplementary file 5 — Supplementary Material 5: Appendix 5. Overview of the Qualitative Analysis Process. [file 12909_2025_7838_MOESM5_ESM.docx]

**Appendix 5: Overview of the Qualitative Analysis Process**

| *Main Themes* | *Sub-themes* | *Key Codes (Examples)* |
| --- | --- | --- |
| Teaching Modelled as Project Consultation | Pedagogical Approached | - Arrangement for Supervision - Importance of Clear Supervision |
|  | Student Learning Paths | - Peer Supervision Engagement - Group Examination Format |
|  | Merging of Improvement Models | - Appraisal of Model Integration - Preference for a Single Model |
| Improving with Evidence | Five Elements of Improvement Work | - End-user Involvement - Barriers to Implementation |
|  | Connecting Improvement needs and Evidence | - Exam Task Structure - Linking Evidence to Improvement Measures |
| Qualifying for Implementation Work | Emphasising the Implementation Phase | - Clinical Practice Applications - Barrier in implementation |
|  | Improvement Competence Important to All Health Workers | - Acquaintance with Improvement Projects - Focus on Evidence-based Practice |
