# Supplementary material for: Integrating quality improvement, evidence-based practice, and knowledge translation into a health Sciences masters’ programme: a mixed methods study
Source: BMC Med Educ. 2025 Oct 14;25:1420. doi: 10.1186/s12909-025-07838-9 (PMC12522345; doi:10.1186/s12909-025-07838-9)
Supplement: Supplementary file 6 — Supplementary Material 6: Appendix 6. Aims in Student Projects Made Available for Analysis. [file 12909_2025_7838_MOESM6_ESM.docx]

**Appendix 6: Aims in Student Projects Made Available for Analysis**

- Improved parental information about infant asymmetry in municipal health services
- Implementing the Olweus-programme in order to prevent bullying at a school
- In the school health service, give more attention to siblings in families with disabled children
- Improve the standardised 8th-grade interview in the school health services
- Increase participation in organised sports among immigrant girls in an urban community
- Improve patient satisfaction in physiotherapy treatment for vulvodynia
- Attention to biology in hospital rotation plans for nurses in order to reduce medication errors
- Strengthen the use of evidence-based practice in municipal physiotherapy services
- Better use of information from questionnaires routinely given to patients during admission to a rehabilitation facility
- In a hospital ward, improve information about the long-term effects of cancer treatment
- Decrease hospital re-admission by improving long-term follow-up for chronic obstructive pulmonary disease patients
- Prevent malnutrition among older adults receiving municipal home care services
- Prevent hospital re-admission of multimorbid elderly
- Implementation of fall-preventing routines in-home care services
