# Supplementary material for: Integrating quality improvement, evidence-based practice, and knowledge translation into a health Sciences masters’ programme: a mixed methods study
Source: BMC Med Educ. 2025 Oct 14;25:1420. doi: 10.1186/s12909-025-07838-9 (PMC12522345; doi:10.1186/s12909-025-07838-9)
Supplement: Supplementary file 7 — Supplementary Material 7: Part I. Mixed Methods Appraisal Tool (MMAT), version 2018. [file 12909_2025_7838_MOESM7_ESM.docx]

**Part I: Mixed Methods Appraisal Tool (MMAT), version 2018**

| **Category of study designs** | **Methodological quality criteria** | **Responses** | | | |
| --- | --- | --- | --- | --- | --- |
|  |  | Yes | No | Can’t tell | Comments |
| Screening questions  (for all types) | S1. Are there clear research questions? | X |  |  | Revised and clearly stated on page 5. |
|  | S2. Do the collected data allow to address the research questions? | X |  |  | Clear link between RQ and self-reported data |
|  |  | | | | |
| 1. Qualitative | 1.1. Is the qualitative approach appropriate to answer the research question? | X |  |  |  |
|  | 1.2. Are the qualitative data collection methods adequate to address the research question? | X |  |  | The combination of interviews and exam papers provides varied data |
|  | 1.3. Are the findings adequately derived from the data? | X |  |  |  |
|  | 1.4. Is the interpretation of results sufficiently substantiated by data? | X |  |  | Themes developed are summed up and supported by citations |
|  | 1.5. Is there coherence between qualitative data sources, collection, analysis and interpretation? | X |  |  | Both data types addressed both in analysis and discussion |
| 2. Quantitative randomized controlled trials | 2.1. Is randomization appropriately performed? |  |  |  | Not applicable (NA) |
|  | 2.2. Are the groups comparable at baseline? |  |  |  | NA |
|  | 2.3. Are there complete outcome data? |  |  |  | NA |
|  | 2.4. Are outcome assessors blinded to the intervention provided? |  |  |  | NA |
|  | 2.5 Did the participants adhere to the assigned intervention? |  |  |  | NA |
| 3. Quantitative non-randomized | 3.1. Are the participants representative of the target population? | X |  |  | Addressed on page 6 |
|  | 3.2. Are measurements appropriate regarding both the outcome and intervention (or exposure)? | X |  |  |  |
|  | 3.3. Are there complete outcome data? |  | X |  | Addressed on page 11, 12 |
|  | 3.4. Are the confounders accounted for in the design and analysis? |  | X |  | Not all, addressed on page 23. |
|  | 3.5. During the study period, is the intervention administered (or exposure occurred) as intended? | X |  |  |  |
| 4. Quantitative descriptive | 4.1. Is the sampling strategy relevant to address the research question? | X |  |  |  |
|  | 4.2. Is the sample representative of the target population? |  | X |  | Low response rate, page 12 |
|  | 4.3. Are the measurements appropriate? | X |  |  |  |
|  | 4.4. Is the risk of nonresponse bias low? |  | X |  | Low response rate, page 12 |
|  | 4.5. Is the statistical analysis appropriate to answer the research question? | X |  |  |  |
| 5. Mixed methods | 5.1. Is there an adequate rationale for using a mixed methods design to address the research question? | X |  |  | Addressed on page 5 and 6 |
|  | 5.2. Are the different components of the study effectively integrated to answer the research question? | X |  |  |  |
|  | 5.3. Are the outputs of the integration of qualitative and quantitative components adequately interpreted? | X |  |  | Table 3 on page 21 |
|  | 5.4. Are divergences and inconsistencies between quantitative and qualitative results adequately addressed? | X |  |  | Inconsistencies were not observed |
|  | 5.5. Do the different components of the study adhere to the quality criteria of each tradition of the methods involved? | X |  |  |  |
